# Two novel mouse models mimicking minor deletions in 22q11.2 deletion syndrome revealed the contribution of each deleted region to psychiatric disorders

**DOI:** 10.1186/s13041-021-00778-7

**Published:** 2021-04-12

**Authors:** Ryo Saito, Chika Miyoshi, Michinori Koebis, Itaru Kushima, Kazuki Nakao, Daisuke Mori, Norio Ozaki, Hiromasa Funato, Masashi Yanagisawa, Atsu Aiba

**Affiliations:** 1 Laboratory of Animal Resources, Center for Disease Biology and Integrative Medicine, Graduate School of Medicine, The University of Tokyo, 7-3-1 Hongo, Bunkyo-ku, Tokyo, 113-0033 Japan; 2 International Institute for Integrative Sleep Medicine (WPI-IIIS), University of Tsukuba, 1-1-1 Tennodai, Tsukuba, 305-8575 Japan; 3 Department of Psychiatry, Nagoya University Graduate School of Medicine, 65 Tsurumai-cho, Showa-ku, Nagoya, 466-8550 Japan; 4 Medical Genomics Center, Nagoya University Hospital, 65 Tsurumai-cho, Showa-ku, Nagoya, 466-8550 Japan; 5 Institute of Experimental Animal Sciences, Graduate School of Medicine, Osaka University, 2-2 Yamadaoka, Suita, Osaka 565-0871 Japan; 6 Brain and Mind Research Center, Nagoya University, 65 Tsurumai-cho, Showa-ku, Nagoya, 466-8550 Japan

**Keywords:** 22q11.2 deletion syndrome, Mouse models, Rare deletion types, Behavioral analysis, Sleep analysis

## Abstract

22q11.2 deletion syndrome (22q11.2DS) is a disorder caused by the segmental deletion of human chromosome 22. This chromosomal deletion is known as high genetic risk factors for various psychiatric disorders. The different deletion types are identified in 22q11.2DS patients, including the most common 3.0-Mb deletion, and the less-frequent 1.5-Mb and 1.4-Mb deletions. In previous animal studies of psychiatric disorders associated with 22q11.2DS mainly focused on the 1.5-Mb deletion and model mice mimicking the human 1.5-Mb deletion have been established with diverse genetic backgrounds, which resulted in the contradictory phenotypes. On the other hand, the contribution of the genes in 1.4-Mb region to psychiatric disorders is poorly understood. In this study, we generated two mouse lines that reproduced the 1.4-Mb and 1.5-Mb deletions of 22q11.2DS [*Del(1.4 Mb)/*+ and *Del(1.5 Mb)/*+] on the pure C57BL/6N genetic background. These mutant mice were analyzed comprehensively by behavioral tests, such as measurement of locomotor activity, sociability, prepulse inhibition and fear-conditioning memory. *Del(1.4 Mb)/*+ mice displayed decreased locomotor activity, but no abnormalities were observed in all other behavioral tests. *Del(1.5 Mb)/*+ mice showed reduction of prepulse inhibition and impairment of contextual- and cued-dependent fear memory, which is consistent with previous reports. Furthermore, apparently intact social recognition in *Del(1.4 Mb)/*+ and *Del(1.5 Mb)/*+ mice suggests that the impaired social recognition observed in *Del(3.0 Mb)/*+ mice mimicking the human 3.0-Mb deletion requires mutations both in 1.4-Mb and 1.5 Mb regions. Our previous study has shown that *Del(3.0 Mb)/*+ mice presented disturbance of behavioral circadian rhythm. Therefore, we further evaluated sleep/wakefulness cycles in *Del(3.0 Mb)/*+ mice by electroencephalogram (EEG) and electromyogram (EMG) recording. EEG/EMG analysis revealed the disturbed wakefulness and non-rapid eye moving sleep (NREMS) cycles in *Del(3.0 Mb)/*+ mice, suggesting that *Del(3.0 Mb)/*+ mice may be unable to maintain their wakefulness. Together, our mouse models deepen our understanding of genetic contributions to schizophrenic phenotypes related to 22q11.2DS.

**Supplementary Information:**

The online version contains supplementary material available at 10.1186/s13041-021-00778-7.

## Introduction

The 22q11.2 deletion syndrome (22q11.2DS) is the most common chromosomal microdeletion disorder in humans, with an estimated incidence of 1 in 1000–4000 live births [1–3]. Individuals with 22q11.2DS show multisystemic physical symptoms including cardiac malformations, velopharyngeal insufficiency, congenital hypocalcemia, thymus hypoplasia, immune deficiency and urogenital malformations. Moreover, 22q11.2DS is known to increase the risk of developing a variety of psychiatric and developmental disorders including schizophrenia, intellectual disability, autism spectrum disorder and attention deficit hyperactivity disorder (ADHD) [4]. The overall penetrance for these psychiatric disorders reaches 100% (95% confidence interval: 60–100) in 22q11.2DS [5].

Most (~ 90%) of individuals affected with 22q11.2DS have a 3.0-Mb hemizygous deletion on chromosome 22q11.2 [6–10]. Less frequently, approximately 7% of the patients have a proximal half of the 3.0-Mb deletion (referred to as 1.5-Mb deletion), and fewer patients have the distal half of the 3.0-Mb deletions (referred to as 1.4-Mb deletion) [7, 9, 11]. These chromosomal deletions occur as a result of meiotic unequal non-allelic homologous recombination (NAHR) induced by multiple segmental duplications (low copy repeats: LCRs), termed LCR22s. The several large size LCR22s (referred to as LCR22A, B, C and D) located in the 3.0-Mb region on chromosome 22q11.2. The most frequent 3.0-Mb deletion is caused by NAHR between LCR22A and LCR22D, and the 1.5-Mb deletion and 1.4-Mb deletion are caused by NAHR between LCR22A and B and between LCR22B and D, respectively [12–14].

Forty-five protein cording genes are located within the 3.0-Mb deleted region and 37 of them are conserved in the mouse chromosome 16qA13, making it possible to generate a mouse model of 22q11.2DS. To date, five lines of 22q11.2DS mouse model have been generated [15–19], four of which mimicked the 1.5-Mb deletion (LCR22A–B deletion). Thus, the contributions of the genes located on the remaining 1.4-Mb region (LCR22B–D in 1.4-Mb deletion) to this syndrome are poorly understood. Although this 1.4-Mb deletion is included in 3.0-Mb deletion of *Del(3.0 Mb)/*+ mouse model mimicking the most common 3.0-Mb deletion in the human 22q11.2 locus, the influence of 1.4-Mb deletion alone on psychiatric phenotypes has not been studied in this model [19]. Individuals with this 1.4-Mb deletion including LCR22B–D and LCR22C–D deletion have been diagnosed as ADHD, anxiety disorder, developmental delay and intellectual disability [6]. Therefore, it is possible that key genes located in this 1.4-Mb deleted region are responsible for the development of psychiatric disorders. Furthermore, repeated behavioral analyses of the other 22q11.2DS mouse models show contradicting results depending on the experimental paradigms. In this regard, it is known that a difference in the genetic background gives rise to variable phenotypic expression, making direct comparisons among different models challenging [20, 21]. For example, previous 1.5-Mb deletion mouse models, except for *Df(h22q11)/*+, were generated by introducing the deletion into the donor chromosome from different strains. Even though they were backcrossed with C57BL/6 strain afterwards, the donor chromosome from original strains remains, leaving skepticism about direct comparisons of phenotypes and accurate phenotypic evaluation of each gene deletion. The advent of CRISPR/Cas9 system enabled the generation of mutants in a pure genetic background by CRISPR/Cas9-mediated genome rearrangement [22–25]. In the present study, we generated two mouse lines that reproduced the 1.4-Mb deletion and 1.5-Mb deletion in 22q11.2DS and characterized their behavioral phenotypes. Furthermore, our previous study has shown the deficits of PPI and disturbance of behavioral circadian rhythm in *Del(3.0 Mb)/*+ mice [19]. For further analysis, we evaluated the rescuing effects of the anti-psychotic drug on the reduced PPI and sleep/wakefulness cycles related to circadian rhythm using *Del(3.0 Mb)/*+ mice.

## Methods

### Animals

Animals were housed under a 12 h light/dark cycle (light on at 8:00, off at 20:00) with maintained temperature (23 ± 1 °C) and humidity (55 ± 10%) and ad libitum access to food and water. Animal experiments were approved by the Institutional Animal Care and Use Committee of The University of Tokyo and conducted in accordance with the guideline of The University of Tokyo.

### Preparation of single-guide RNAs (sgRNAs), *Cas9* mRNA and single-stranded oligodeoxyribonucleotide (ssODN)

To design sgRNA with smaller number of off-target sites, CRISPRdirect software was used (http://crispr.dbcls.jp; [26]). Two pairs of sgRNAs positioned on either endpoint of the deletion were designed. The protospacer sequences of sgRNAs were listed in Additional file 1 (Table S1). These protospacers were cloned into *Bsa*I-pDR274 vector (Addgene #42250). The sgRNAs were transcribed in vitro using the *Dra*I-digested pDR274 vectors as a template and the MEGAshortscript T7 kit (Ambion, CA, USA) and purified using MEGAclear kit (Ambion). The *Cas9* mRNA was transcribed in vitro using a MessageMax T7 ARCA-Capped Message mRNA transcription kit (Cellscript, WI, USA), polyadenylated with an A-plus Poly(A) Polymerase Tailing kit (Cellscript) and purified using MEGAclear kit (Ambion). The ssODNs designed to bridge the deletion endpoints were 120 nucleotides in length and positioned directly adjacent to the most external sgRNA site. The sequences of ssODNs were listed in Additional file 2 (Table S2).

### Microinjection of *hCas9* mRNA, sgRNAs and ssODN

*Cas9* mRNA, sgRNAs and ssODN were delivered by microinjection to the cytoplasm of C57BL/6N (CLEA Japan Inc., Tokyo, Japan) fertilized eggs as described previously [19]. Briefly, fifty ng/μL of *hCas9* mRNA, 25 ng/μL of sgRNA (each) and 100 ng/μL of ssODN were mixed in RNase-free water and microinjected. Survived microinjected embryos were cultured in modified Whitten’s medium (mWM) until they reached the 2-cell stage. Injected embryos were transferred into oviducts of 0.5-day-post-coitum recipients (ICR, CLEA Japan Inc.).

### Genotyping of genome engineered mice by PCR assay

Genomic DNA was extracted from mouse tail tips to serve as templates for genotyping PCR assay. PCR products were amplified with Ex *Taq* DNA polymerase (Takara Bio Inc., Shiga, Japan). The PCR primers for genotyping of *Del(1.4 Mb)/*+ mice were as follows: 5′-AACCACAGGGGTGGAAAGTC-3′ and 5′-TGCTAGCCTGCATCGTAAGG-3′ (552 bp product, the deletion band); 5′-CACTTGTCAACTGACTACTGTTTG-3′ and 5′-GAGCCAGGTCTAGGAACGTC-3′ (654 bp product, the internal control). The following primers were used for genotyping of *Del(1.5 Mb)/*+ mice: 5′-CTAAGGAATGGTTCCGGCCA-3′ and 5′-TTTCACGGAGGCGGTATTCA-3′ (424 bp product, the deletion band); 5′-GAGAAAGTGGGTGGGAAGGC-3′ and 5′-GTCCCTCGCCACAGTCATAA-3′ (532 bp product, the internal control). The reaction for detection of 1.4-Mb deletion was conducted under the following conditions: initial denaturation at 98 °C for 2 min, 30 cycles of melting at 98 °C for 30 s, annealing at 64 °C for 30 s, and extension at 72 °C for 30 s, with additional extension at 72 °C for 2 min at the end. The condition of 1.5-Mb deletion was as follows: 98 °C for 2 min, 30 cycles of 98 °C for 30 s, 65 °C for 30 s, and 72 °C for 30 s, with additional extension at 72 °C for 2 min at the end. PCR products were analyzed in 2% agarose gel electrophoresis and the sequences were confirmed by Sanger sequencing (Fasmac Co., Kanagawa, Japan).

### Array comparative genomic hybridization (array CGH) analysis

Array CGH analysis was performed according to previously reported methods [10]. In briefly, an Agilent SurePrint G3 Mouse CGH 4 × 180 K Microarray (Agilent, Santa Clara, CA, USA) was used according to the manufacturer’s instructions. Copy number variation (CNV) calls were made with Nexus Copy Number software v9.0 (BioDiscovery, El Segundo, CA, USA) using the Fast Adaptive States Segmentation Technique 2 (FASST2) algorithm, which is a hidden Markov model-based approach. The log_2_ ratio threshold for copy number loss (or deletion) was set at − 0.4. The significance threshold to adjust the sensitivity of the segmentation algorithm was set at 1 × 10^−6^, and at least five contiguous probes were required for CNV calls. Genomic locations are reported in NCBI Build 37/UCSC mm9 coordinates.

### Quantitative RT-PCR

Total RNA was isolated from hippocampi of male mice using miRNeasy Mini Kit (QIAGEN, MD, USA). For quantification of mRNA, first strand cDNA was synthesized using PrimeScript RT reagent Kit (Perfect Real Time) (Takara Bio Inc.). Briefly, 300 ng of total RNA was reverse transcribed using 25 pmol of oligo dT primer and 50 pmol of random 6 mer in 10 μL reaction. The resultant cDNA was diluted at 1:30 ratio in TE. All samples within an experiment were reverse transcribed at the same time. All real-time PCR reactions were performed using the PowerUp SYBR Green Master Mix (Applied Biosystems, CA, USA) and the StepOnePlus Real-Time PCR system (Applied Biosystems). The experiments were conducted in triplicate for each data point. The relative quantification in gene expression was determined using ∆∆Ct method [27]. The sequences of the primers are listed in Additional file 3 (Table S3).

### Behavioral tests

All behavioral tests were carried out with male mice at the age of 8–11 weeks. We used *Del(1.4 Mb)/*+ and *Del(1.5 Mb)/*+ lines which were backcrossed to C57BL/6N for 3 generations. *Del(3.0 Mb)/*+ mice was backcrossed to C57BL/6N for 4 generations. Prior to each experiment, mice were placed in the testing room for at least 1 h to acclimate to the experimental environments. The experimenter was blind to genotype throughout the experimental procedures. All apparatus used in behavioral tests were cleaned with 70% ethanol and wiped with paper towels between each session. For the characterization of behavioral phenotypes in *Del(1.4 Mb)/*+ and *Del(1.5 Mb)/*+ mice, we conducted a series of behavioral tests consisted of the following testing paradigms: (1) open field test, (2) five-trial social interaction test, (3) prepulse inhibition, and (4) contextual and cued fear conditioning test. We set at least 1 day of rest between each testing paradigm.

### Open field test

Mice were placed in the center of the open field arena (diameter: 75 cm, height: 35 cm) and were allowed to explore freely the arena for the following 10 min under moderately light conditions (15 lx). Their movement was recorded with a camera mounted above the arena, and their activity was measured automatically using Smart V3.0 tracking software (Panlab, Barcelona, Spain). The open field was divided into an inner circle (diameter: 50 cm), and an outer area surrounding the inner circle. Measurements included total distance moved, time spent in the outer zone and number of transitions between inner and outer section.

### Five-trial social interaction test

Five-trial direct social interaction test was performed as described previously [19]. Subject mice were placed individually into home cage (45 cm × 28 cm × 16 cm) for 1 h before starting test under the moderately light conditions (15 lx). A juvenile intruder mouse (5-week-old) was introduced into the subject mouse’s home cage. The subject mouse was exposed to the same intruder mouse for 5 min over 4 trials with an inter-trial-interval of 30 min. During the fifth trial, the subject mouse was exposed to a novel intruder mouse (5-week-old). The time spent in social interaction (close following, inspection, anogenital sniffing and other social body contacts) was recorded.

### Prepulse inhibition (PPI)

The prepulse inhibition (PPI) test was carried out by using SR-Lab system (San Diego Instruments, San Diego, CA, USA) as described previously [19]. After each mouse was placed in an enclosure (12.7 cm, 3.8 cm inner diameter) under moderately bright light condition (180 lx), they were acclimated for 10 min in the presence of background white noise (65 dB). The movement of the animal in the startle chamber was measured by a piezoelectric accelerometer mounted under the enclosure at the sampling rate of 1 kHz. Individual mouse received 20 startle trials, 10 no-stimulus trials and 40 PPI trials. The inter-trial interval was between 10 and 20 s. The startle trial consisted of a single 120 dB white noise burst lasting 40 ms. PPI trials consisted of a prepulse (20 ms burst of white noise at 69, 73, 77 or 81 dB intensity) followed by the startle stimulus (120 dB, 40 ms white noise) 100 ms later. Each of the four prepulse trials (69, 73, 77 or 81 dB) was carried out 10 times. Five consecutive startle trials were presented at the beginning and end of the session. The remainder of sixty different trials were performed pseudorandomly to ensure that each trial was done 10 times and that no two consecutive trials were identical. During the session, 65 dB background white noise was continually present. The largest amplitude in the recording window was taken as the startle amplitude for the trial. Basal startle amplitude was determined as the mean amplitude of the 10 startle trials. PPI (%) was calculated as follows: 100 × (pulse-alone response − prepulse-pulse response)/pulse-alone response, in which prepulse-pulse response was the mean of the 10 PPI trials (69, 73, 77 or 81 dB) and pulse-alone response was the basal startle amplitude.

### Drug administration

Haloperidol Solution (5.0 mg/mL) was obtained from Sumitomo Dainippon Pharma Co., Ltd. (Tokyo, Japan) and was diluted in saline. Haloperidol (0.3 mg/kg) was administrated intraperitoneally (i.p.) 30 min before PPI experiment.

### Contextual and cued fear conditioning test

The fear conditioning test was conducted using ImageJ FZ1 (O’Hara & Co., Ltd., Tokyo, Japan) as described previously [19]. The conditioning chamber was a square arena (10 cm × 10 cm × 10 cm) with clear Plexiglas walls and a metal grid floor connected to a circuit board that delivered electric shocks to the metal grid. A video camera was set in front of the cage to record the behavior. In the conditioning session, mice were individually placed into the conditioning chamber and allowed to explore freely for 3 min. After 3 min exploratory period, each mouse was exposed to two tone-footshock pairings (tone, 30 s; footshock, 2 s, 0.8 mA at the termination of the tone; separated by 1 min intertrial interval). One min after the second footshock, the mouse was returned to its home cage. Twenty-four h after conditioning, the context-dependent test was performed, in which each mouse was placed back into the conditioning chamber, and the freezing response was measured for 6 min in the absence of the conditioned stimulus. Forty-eight h after the footshock, each mouse was tested for auditory (tone) fear conditioning in a novel opaque chamber. Different environmental cues (e.g. light condition and background noise) were provided in the novel chamber. Mice were tested in the novel chamber for a 3 min baseline period (pre-tone) followed by another 3 min for the conditioning tone during which the tone was presented persistently for 3 min. Total freezing rate was measured as an index of fear memory. Motionless bouts lasting more than 2 s were considered as freeze.

### Electroencephalogram (EEG) and electromyogram (EMG) analysis

Sleep analysis was performed as described previously [28]. Eight- to ten-week-old mice were subjected to EEG/EMG electrode implantation surgery. The surgery was performed under isoflurane anesthesia (4% for induction, 2% for maintenance). The scalp was incised along the midline to expose the cranium. Four holes were generated on the skull using 1.0-mm drill bits (anteroposterior: 0.50 mm, lateral: ± 1.27 mm and anteroposterior: − 4.53 mm, lateral: ± 1.27 mm from bregma). The four electrode pins were lowered onto the dura under stereotaxic control (David Kopf Instruments, #940/926) and fixed using dental cement (3 M ESPE, Ketac Cem Aplicap). Subsequently, two EMG wires were inserted into the neck extensor muscles and covered with dental cement. A 6-pin header dissected from 2 × 40 pin header (Useconn Electronics Ltd., #PH-2 × 40SG) was inserted to the top of the EEG/EMG electrode into close the holes of the insulator.

Seven days after surgery, the mice were attached to a tether cable and singly housed in a recording cage (19.1 × 29.2 × 12.7 cm). The tether cable was hung by a counterbalanced lever arm (11.4 cm-long, Instech Laboratories, #MCLA) that allows the mice to move freely. All mice were allowed at least 5 days of recovery from surgery and habituation to the recording conditions for at least 4 days. The floor of the cage was covered with aspen chips and nest materials. To examine sleep/wake behavior under baseline conditions, the EEG/EMG signal was recorded for three consecutive days from the onset of the light phase.

EEG/EMG signals were amplified, filtered (EEG: 0.5–100 Hz; EMG: 0.5–300 Hz) with a multi-channel amplifier (NIHON KODEN, #AB-611J), and digitized at a sampling rate of 250 Hz using an analogue-to-digital converter (National Instruments, #PCI-6220) with LabVIEW software (National Instruments). The EEG/EMG data were visualized and semi-automatically analyzed by MATLAB-based software followed by visual inspection. Each 20-s epoch was staged into wakefulness, non-rapid eye movement (NREM) sleep and REM sleep. Wakefulness was scored based on the presence of low amplitude, fast EEG activity and high amplitude, variable EMG activity. NREM sleep was characterized by high amplitude, delta (1–4 Hz)-frequency EEG waves and a low EMG tonus, whereas REM sleep was staged based on theta (6–9 Hz)-dominant EEG oscillations and EMG atonia. The total time spent in wakefulness, NREM sleep, and REM sleep were derived by summing the total number of 20-s epochs in each state. Mean episode durations were determined by dividing the total time spent in each state by the number of episodes of that state. Epochs that contained movement artifacts were included in the state totals but excluded from the subsequent spectral analysis. EEG signals were subjected to fast Fourier transform analysis from 1 to 30 Hz with 1-Hz bins using MATLAB-based custom software. The EEG power density in each frequency bin was normalized to the sum of 16–30 Hz in all sleep/wake state.

### Statistical analysis

The significance of differences (*p* < 0.05) was assessed by two-tailed Welch’s *t*-test for comparison of two groups. In multiple comparison, the nonparametric Kruskal–Wallis test was used because the homogeneity of variance was violated by Bartlett test. For the analysis of variance (ANOVA) with two factors (two-way repeated measure ANOVA), normality of sample distribution was assumed, and followed by Bonferroni post hoc analysis. Differences in frequency distribution were evaluated using the Kolmogorov–Smirnov test. All data are expressed as the mean ± standard error of the mean (SEM). The detailed statistics methods and values were described at Additional file 4 (Table S4).

## Results

### Generation of *Del(1.4 Mb)/*+ and *Del(1.5 Mb)/*+ mice

**Fig. 1:**
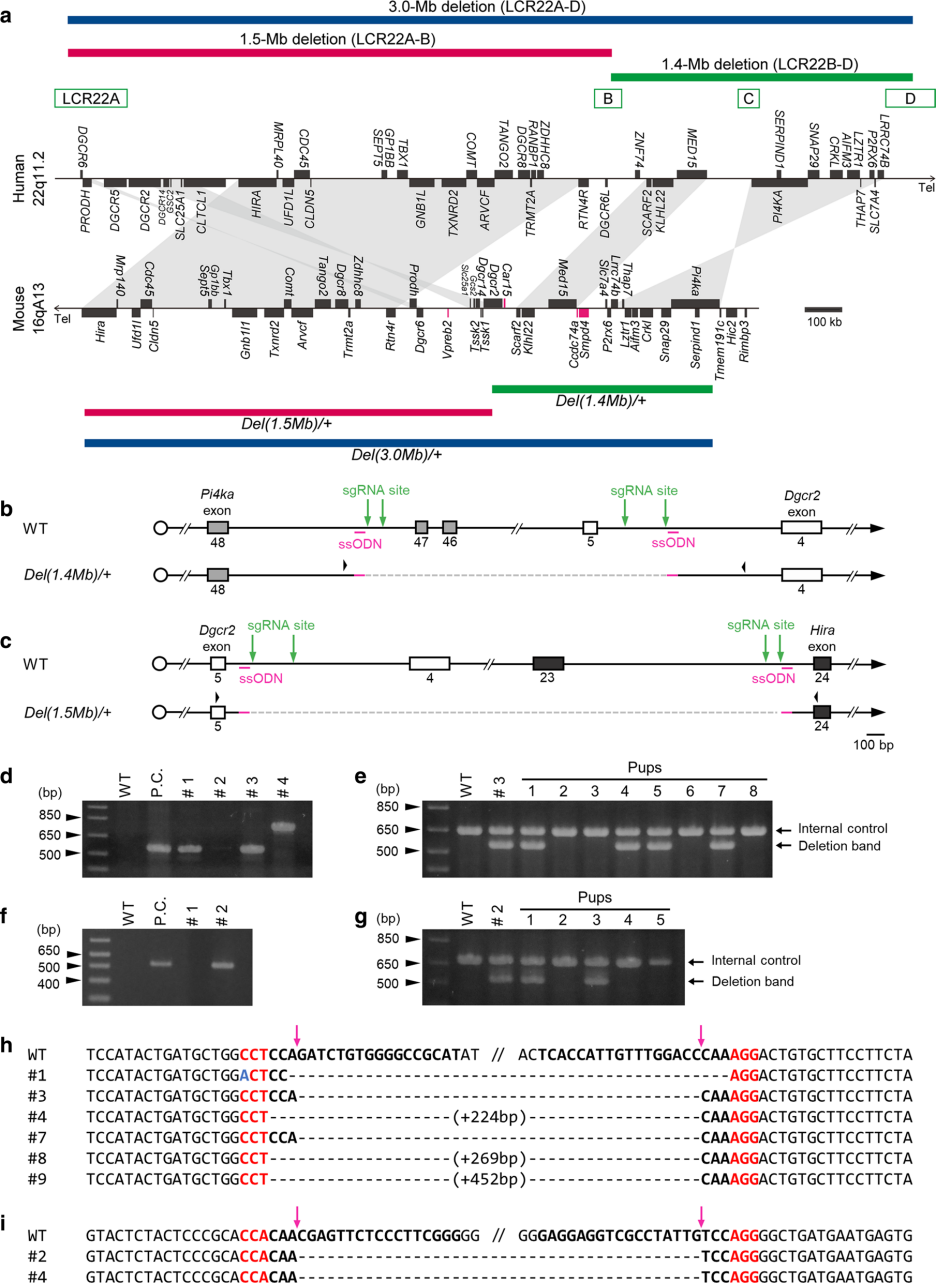
Generation of *Del(1.4 Mb)/*+ and *Del(1.5 Mb)/*+ mice. **a** Schematic diagram showing the mouse chromosome 16qA13 that is syntenic region of human chromosome 22q11.2. Green boxes represent low copy repeats (LCRs). Each black square represents one gene which is conserved between human and mouse. Pink squares represent non-conserved genes. Blue, red, and green horizontal bars indicate the human hemizygous genomic deletions (3.0-Mb, 1.5-Mb and 1.4-Mb, respectively) and syntenic mouse genomic regions [*Del(3.0 Mb)/*+, *Del(1.5 Mb)/*+ and *Del(1.4 Mb)/*+]. **b** Schematic illustration of wild-type (WT) and *Del(1.4 Mb)/*+ alleles. Green arrows, sgRNA sites; pink bars, bridging single-stranded oligodeoxyribonucleotides (ssODNs); gray squares, *Pi4ka* exons; white squares, *Dgcr2* exons; black arrowheads, PCR primers; dashed line, deleted region in the *Del(1.4 Mb)/*+ allele. **c** Schematic illustration of WT and *Del(1.5 Mb)/*+ alleles. Green arrows, sgRNA sites; pink bars, bridging ssODNs; white squares, *Dgcr2* exons; black squares, *Hira* exons; black arrowheads, PCR primers; dashed line, deleted region in the *Del(1.5 Mb)/*+ allele. **d** A representative result of genotyping PCR analysis of *Del(1.4 Mb)/*+ founder candidates. P.C., positive control. **e** Genotyping analysis of N1 offspring of *Del(1.4 Mb)/*+ founder × WT crossing. **f** A representative result of genotyping PCR analysis of *Del(1.5 Mb)/*+ founder candidates. P.C., positive control. **g** Genotyping analysis of N1 offspring of *Del(1.5 Mb)/*+ founder × WT crossing. **h** The nucleotide sequencing analysis of PCR-amplified fragments around the deletion junction in *Del(1.4 Mb)/*+ founder candidates. sgRNA sites are indicated in bold and protospacer adjacent motif (PAM) sites in red. A mismatch nucleotide is indicated in blue. The expected sgRNA-guided cutting sites are indicated by pink arrows. **i** Deletion junction sequences of *Del(1.5 Mb)/*+ founder candidates. sgRNA sites are indicated in bold and PAM sites in red. The expected sgRNA-guided cutting sites are indicated by pink arrows

To generate two mutant lines with partial deletions in 22q11.2DS (Fig. 1a), we conducted CRISPR/Cas9-mediated chromosome editing using C57BL/6N mouse zygotes as previously described [19, 22]. To introduce the deletion between *Phosphatidylinositol 4-kinase alpha* (*Pi4ka*) and *DiGeorge syndrome critical region 2* (*Dgcr2*) on mouse chromosome 16, we designed a pair of sgRNAs on each target locus of *Pi4ka* intron 47 and *Dgcr2* intron 4 and an ssODN to bridge two Cas9 cleavage sites directly (Fig. 1b). Then, two pairs of sgRNAs, *hCas9* mRNA and a bridging ssODN were co-injected into the cytoplasm of 838 C57BL/6N zygotes, and 432 viable 2-cell embryos were transplanted into recipient ICR female mice (Additional file 5: Table S5). We obtained a total of 23 pups from manipulated embryos. Nine out of 23 pups died before weaning. Genotyping PCR assay of 14 viable founder candidates were conducted (Fig. 1d) and the deletion were confirmed by direct sequencing of the PCR products (Fig. 1h). Six offspring harbored the desired structural variants (Additional file 5: Table S5). We used founder #3 to establish a mutant line [hereinafter referred to as *Del(1.4 Mb)/*+]. The deletion allele of the founder was successfully transmitted through the germline (Fig. 1e).

To generate a mouse with a deletion in the targeted region from *Dgcr2* to *Histone Cell Cycle Regulator* (*Hira*) [hereinafter referred to as *Del(1.5 Mb)/*+], we used a pair of sgRNAs on each locus of *Dgcr2* intron 4 and *Hira* intron 23 and an ssODN bridging the deleted region (Fig. 1c). We obtained a total of 10 pups from manipulated embryos and 2 pups harbored the desired mutation (Fig. 1f, i and Additional file 5: Table S5). The deletion allele of the founder #2 was successfully transmitted through the germline (Fig. 1g).

### The decrease in genomic copy number and mRNA expression of genes within target regions in *Del(1.4 Mb)/*+ and *Del(1.5 Mb)/*+ mice

**Fig. 2:**
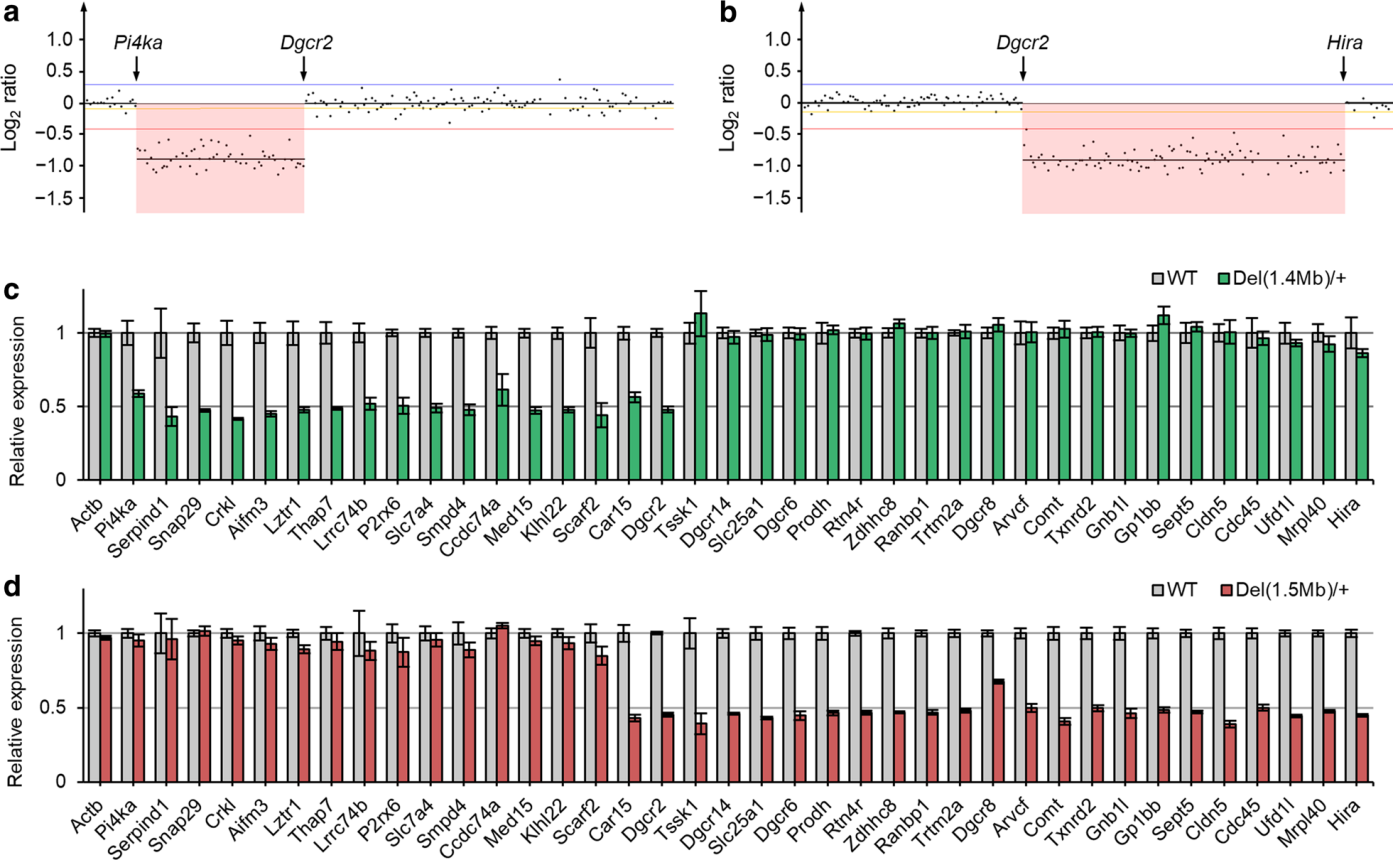
Detection of DNA copy number changes and quantification of mRNA expression in the brain. **a**, **b** Array CGH profiles of chromosome 16qA13 from N1 *Del(1.4 Mb)/*+ (**a**) and *Del(1.5 Mb)/*+ mice (**b**) showing the decrease in copy number of targeting regions (pink). Array CGH data are displayed in a magnified view. **c** Quantitative RT-PCR analysis of mRNA from the hippocampi of WT (n = 5) and *Del(1.4 Mb)/*+ (n = 5) mice. **d** Quantitative RT-PCR analysis of WT (n = 5) and *Del(1.5 Mb)/*+ (n = 5) mRNA. Data are expressed as means ± SEM

To confirm a genomic copy number loss on chromosome 16qA13 in our two mutant lines, we performed array CGH analysis. The expected decrease in genomic copy number of target region was confirmed in both *Del(1.4 Mb)/*+ and *Del(1.5 Mb)/*+ mice (Fig. 2a and b).

Next, to confirm the decreased expression of the deleted genes in the two 22q11.2DS mouse lines, we performed quantitative RT-PCR. We found that expression of the genes in the deleted region between *Pi4ka* and *Dgcr2* was significantly reduced to approximately 50% in *Del(1.4 Mb)/*+ mice compared with WT littermates (Fig. 2c). In *Del(1.5 Mb)/*+ mice, expression of the genes, except for *Dgcr8*, in the deleted region between *Dgcr2* and *Hira* was reduced to approximately 50% of the expression in WT mice (Fig. 2d). Expression of *Dgcr8* was reduced to 67.6%, possibly due to negative feedback regulation by the Microprocessor complex containing DGCR8 itself [29]. In addition, expression of *Carbonic anhydrase 15* (*Car15*) that is adjacent to *Dgcr2* was reduced to 43.1% in *Del(1.5 Mb)/*+ mice, which is consistent with the previous expression analysis of *Df(h22q11)/*+ mice [30].

### General locomotor activity in *Del(1.4 Mb)/*+ and *Del(1.5 Mb)/*+ mice

**Fig. 3:**
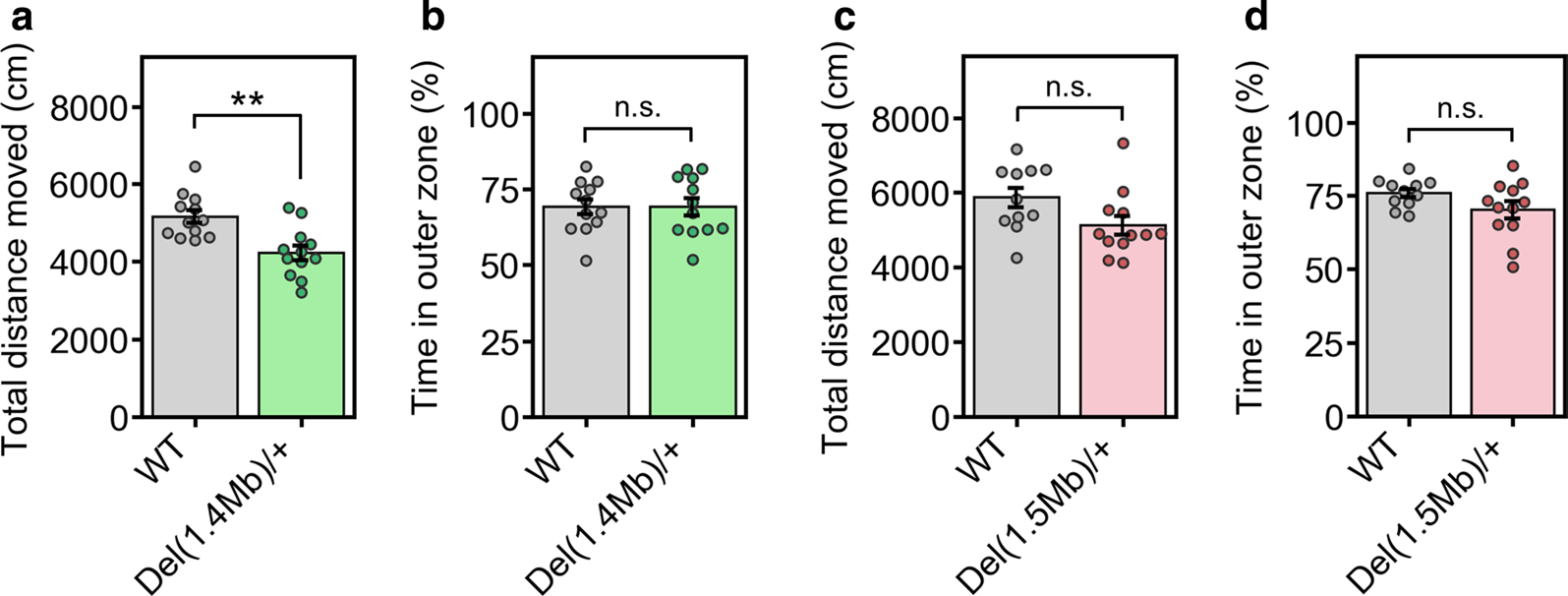
General locomotor activity in the open field test. **a**, **b** Performance in open field test of *Del(1.4 Mb)/*+ mice (n = 12 for WT; n = 12 for *Del(1.4 Mb)/*+ mice). **a** Total distance moved during the 10 min test period. **b** Percentage of time spent in the outer zone. **c**, **d** Performance in open field test of *Del(1.5 Mb)/*+ mice (n = 11 for WT; n = 12 for *Del(1.5 Mb)/*+ mice). **c** Total distance moved during the 10 min test period. **d** Percentage of time spent in the outer zone. Data are expressed as mean ± SEM. *n.s.* not significant; **p < 0.01

Several studies have conducted ethological analysis of the 22q11.2DS mouse models. However, it is not clear whether genes located in 1.4-Mb region contribute to the disease development. Therefore, we characterized the behavioral phenotypes of our two mutant lines. Firstly, to examine the general locomotor activity of *Del(1.4 Mb)/*+ mice, we conducted an open field test for 10 min. The total distance moved of *Del(1.4 Mb)/*+ mice was significantly shorter than that of WT littermates (Fig. 3a), indicating the hypoactivity in *Del(1.4 Mb)/*+ mice. This result is consistent with previous finding of *Del(3.0 Mb)/*+ mice [19]. There was no significant difference in the zone preference between two genotypes (Fig. 3b), indicating that the anxiety-like behavior of *Del(1.4 Mb)/*+ mice was normal. In *Del(1.5 Mb)/*+ mice, we found the decreased mean value of total distance moved as compared to WT littermates, although there is no statistically significant difference between *Del(1.5 Mb)/*+ and WT littermates (Fig. 3c). Also, there is no significant difference in time in outer zone as compared to WT littermate controls (Fig. 3d). Our finding of apparent normal locomotor activity in *Del(1.5 Mb)/*+ mice is consistent with *Df1/*+ mice which are one of the 22q11.2DS mouse models with the 1.5-Mb deletion [31] and inconsistent with other 1.5-Mb deletion models, *Df(16)A*^+*/−*^ and *Lgdel/*+ mice, which have shown hyperactivity [17, 32, 33].

### Sociability and social memory in *Del(1.4 Mb)/*+ and *Del(1.5 Mb)/*+ mice

**Fig. 4:**
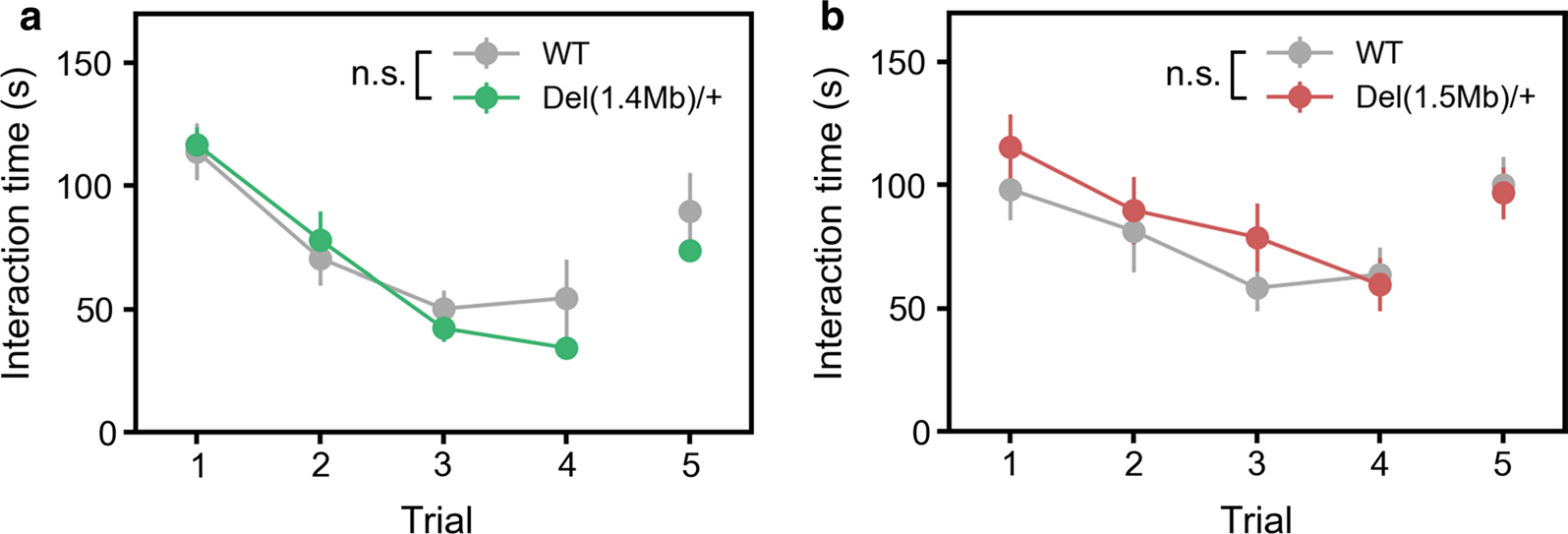
Performance in five-trial direct social interaction test. **a** Performance in five-trial direct social interaction test of *Del(1.4 Mb)/*+ mice (n = 12 for WT; n = 12 for *Del(1.4 Mb)/*+ mice). **b** Performance in five-trial direct social interaction test of *Del(1.5 Mb)/*+ mice (n = 11 for WT; n = 12 for *Del(1.5 Mb)/*+ mice). Data are expressed as mean ± SEM. *n.s.* not significant

Next, to assess sociability and social memory in the two mutant mouse lines, we performed five-trial social interaction test. In this test, a test mouse was exposed to the same intruder mouse for four successive trials (trial 1–4). On the fifth trial, the test mouse was exposed to a novel intruder mouse (trial 5). There was no significant difference in sociability (trial 1–4) and social memory (trial 5) between *Del(1.4 Mb)/*+ mice and WT littermates (Fig. 4a). Similarly, we found no significant difference in the interaction time to intruders between *Del(1.5 Mb)/*+ mice and WT littermates (Fig. 4b).

### Sensorimotor gating of *Del(1.4 Mb)/*+ and *Del(1.5 Mb)/*+ mice

**Fig. 5:**
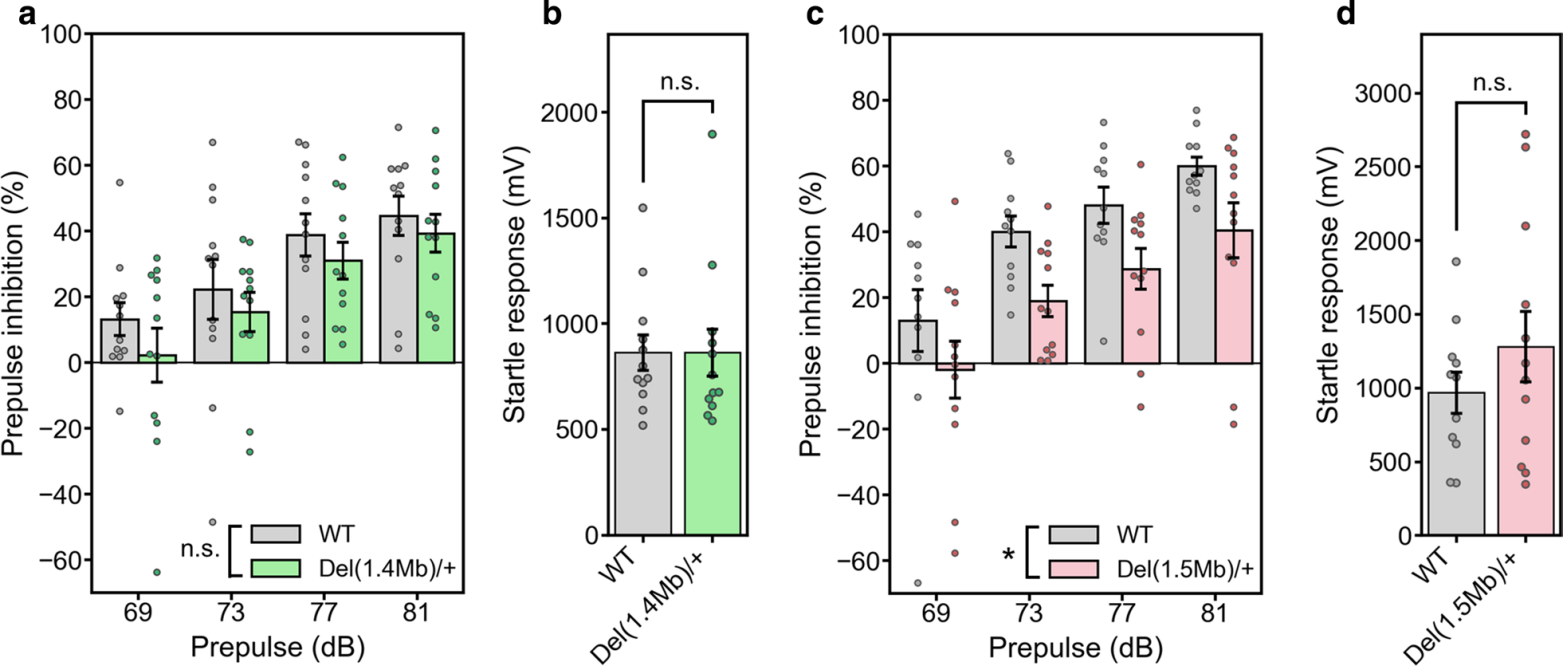
Prepulse inhibition (PPI) analysis of the acoustic startle response. **a** Percentage of PPI in *Del(1.4 Mb)/*+ mice (n = 12 for WT; n = 12 for *Del(1.4 Mb)/*+ mice). **b** Measurement of acoustic startle response to the 120-dB startle stimulus in *Del(1.4 Mb)/*+ mice. **c** Percentage of PPI in *Del(1.5 Mb)/*+ mice (n = 11 for WT; n = 12 for *Del(1.5 Mb)/*+ mice). **d** Measurement of acoustic startle response to the 120-dB startle stimulus in *Del(1.5 Mb)/*+ mice. *n.s.* not significant; *p < 0.05, **p < 0.01, ***p < 0.001

Prepulse inhibition (PPI) of the auditory startle response is often used as a translatable measure of the sensorimotor gating that is considered to be the endophenotypic marker of psychiatric conditions such as schizophrenia [34–37]. Moreover, previous reports performing a PPI analysis showed that reduced PPI is observed in 3.0-Mb and 1.5-Mb deletion mouse models [17, 19, 30–32, 38, 39]. To investigate whether the deletion of genes on the 1.4-Mb region is responsible for the impairment of sensorimotor gating, we conducted auditory PPI analysis in *Del(1.4 Mb)/*+ mice. *Del(1.4 Mb)/*+ mice showed a tendency to decrease PPI, but there was no statistically significant difference between *Del(1.4 Mb)/*+ mice and WT littermates (Fig. 5a). The amplitude of startle response to the loud sound was comparable between *Del(1.4 Mb)/*+ mice and WT littermates (Fig. 5b). Next, we evaluated PPI of *Del(1.5 Mb)/*+ mice. In line with the previous studies, PPI was significantly reduced in *Del(1.5 Mb)/*+ mice compared with WT littermates (Fig. 5c). There was no significant difference in the amplitude of startle response between *Del(1.5 Mb)/*+ mice and WT littermates (Fig. 5d).

### Effects of acute haloperidol administration on PPI deficits in *Del(3.0 Mb)/*+ mice

In our previous study, *Del(3.0 Mb)/*+ mice have exhibited the PPI deficit [19]. In the present study, we investigated whether the PPI deficit observed in *Del(3.0 Mb)/*+ mice was rescued by the typical anti-psychotic drug haloperidol, dopamine D2 receptor antagonist. We found no significant effect of acute haloperidol administration on PPI deficits in *Del(3.0 Mb)/*+ mice (Additional file 6: Figure S1a). Likewise, there was no significant effect of haloperidol on startle response between *Del(3.0 Mb)/*+ groups (Additional file 6: Figure S1b).

### Associative learning and memory of *Del(1.4 Mb)/*+ and *Del(1.5 Mb)/*+ mice

**Fig. 6:**
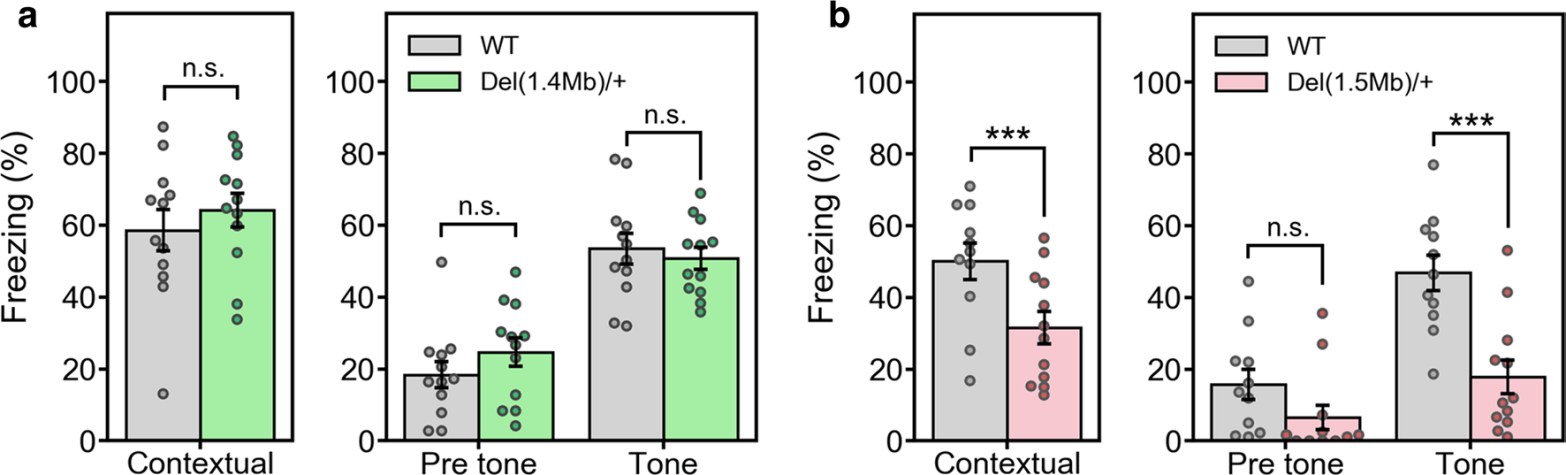
Contextual and cued fear-conditioning test. **a** Fear-conditioning test of *Del(1.4 Mb)/*+ mice (n = 12 for WT; n = 12 for *Del(1.4 Mb)/*+ mice). Context-dependent freezing response (%) towards the tone and foot-shock paring (conditioning) measured 24 h after the initial exposure (left panel). Cued-dependent freezing response (%) measured 48 h after the conditioning (right panel). **b** Fear-conditioning test of *Del(1.5 Mb)/*+ mice (n = 11 for WT; n = 12 for *Del(1.5 Mb)/*+ mice). Context-dependent freezing response (%) (left panel). Cued-dependent freezing response (%) (right panel). Data are expressed as mean ± SEM. *n.s.* not significant; ***p < 0.001

A fear-conditioning test has repeatedly revealed a dysfunction of associative learning and memory in 22q11.2DS mouse models [17, 31–33, 40, 41]. To assess the function of learning and memory in our 22q11.2DS models, we conducted contextual-dependent and auditory cued-dependent fear-conditioning test. There was no significant difference in the freezing time both in contextual-dependent and cued-dependent fear conditioning tests between *Del(1.4 Mb)/*+ and WT littermates (Fig. 6a), indicating the apparently normal learning and memory in *Del(1.4 Mb)/*+ mice. On the other hand, as shown in the previous 1.5-Mb deletion models, *Del(1.5 Mb)/*+ mice showed decreased freezing time both in contextual-dependent and cued-dependent tests (Fig. 6b).

### Sleep/wakefulness analysis of *Del(3.0 Mb)/*+ mice by EEG/EMG recording

**Fig. 7:**
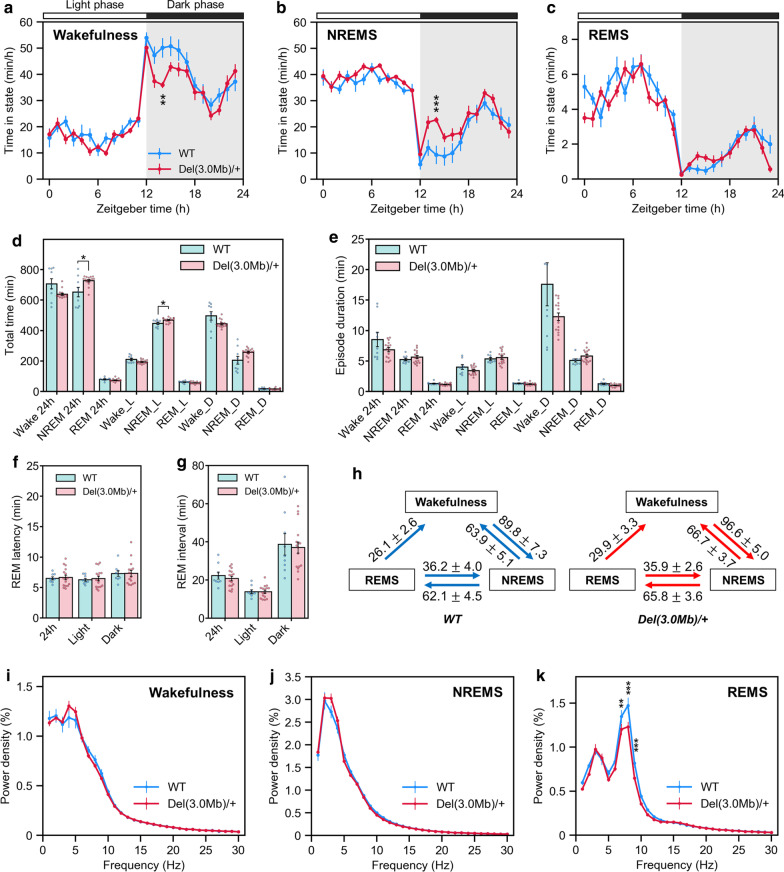
Sleep and wakefulness cycle in *Del(3.0 Mb)/*+ mice. **a**–**c** Circadian variation in wakefulness (**a**), NREM sleep (**b**) and REM sleep (**c**) in *Del(3.0 Mb)/*+ mice (n = 9 for WT; n = 16 for *Del(3.0 Mb)/*+ mice). Data are expressed as minutes per hour spent in each stage, averaged from EEG/EMG recordings during two consecutive 24-h periods. **d** Total time of wakefulness, NREM sleep and REM sleep stages in 24-h period, light period (L) and dark period (D). **e** Episode duration of wakefulness, NREM sleep and REM sleep stages in 24-h period, light period and dark period. **f** REM latency in 24-h period, light period and dark period. **g** REM interval in 24-h period, light period and dark period. **h** Values indicate the number of transitions between wakefulness, NREM sleep and REM sleep per 24 h. **i**–**k** EEG spectral profiles of WT (blue line, n = 9) and *Del(3.0 Mb)/*+ mice (red line, n = 16) during wakefulness (**i**), NREM sleep (**j**) and REM sleep (**k**). The average EEG spectra were normalized to total EEG power from 1 to 32 Hz in 1 Hz bins. Data are expressed as mean ± SEM. *p < 0.05, **p < 0.01, ***p < 0.001

In our previous study, *Del(3.0 Mb)/*+ mice have shown lower behavioral activity in their subject night, suggesting the disturbed intrinsic circadian behavioral rhythm [19]. To investigate the details of their circadian rhythm changes, we conducted electrophysiological analysis of sleep/wakefulness cycles by EEG/EMG recording using *Del(3.0 Mb)/*+ mice. We found that *Del(3.0 Mb)/*+ mice showed no distinct changes in the hourly pattern during the light phase in all sleep and wake stages. However, there was reduction in wakefulness and increased NREMS time during the first few hours immediately after the start of dark period with statistical significance at Zeitgeber time (ZT) 14 (Fig. 7a and b), but no difference in the REMS between *Del(3.0 Mb)/*+ mice and WT (Fig. 7c). These changes in the hourly pattern of wakefulness and NREMS did not reflect the cumulative time spent in each stage during light and dark phases (Fig. 7d). Episode duration of each stage was not significantly different between *Del(3.0 Mb)/*+ and WT littermates (Fig. 7e). There were no significant differences in the REM latency and interval (Fig. 7f and g) and no significant difference in the transition among wakefulness, NREMS and REMS (Fig. 7h). In EEG spectral analysis, EEG power densities during wakefulness and NREMS did not significantly differ between *Del(3.0 Mb)/*+ and WT littermates (Fig. 7i and j). Meanwhile, EEG power density in the theta frequency range (6‒12 Hz) during REMS was significantly reduced in *Del(3.0 Mb)/*+ mice as compared with WT littermates (Fig. 7k).

## Discussion

22q11.2DS is known to be implicated in the onset of various psychiatric disorders. Since 22q11.2 deletion has the highest risk of schizophrenia, this syndrome has been considered as one of the most important genetic risk factors of schizophrenia. In 22q11.2DS, different deletion types have been identified. Approximately 90% of 22q11.2DS patients have the deletion of 3.0-Mb range, and the deletions of 1.5-Mb and 1.4-Mb regions are much less common. To date, most of animal studies associated with 22q11.2DS have focused on the 1.5-Mb deletion. Several genes encoded in the 1.5-Mb region (e.g., *Tbx1*, *Dgcr8*, *Comt*, *Sept5*, and *Prodh*) have been identified as potentially associated with psychiatric disorder-like behavioral phenotypes [42]. The contribution of the 1.4-Mb deletion for the onset of psychiatric disorders has been suggested in clinical studies [7, 9–11], and *Crkl* (*CRK-like Proto-Oncogene, Adaptor Protein*) heterozygous-deficient mice (*Crkl*^+*/−*^) have been analyzed to investigate the contributions of *Crkl* gene to behavioral phenotypes relevant to psychiatric disorders [43]. However, the functional role of other genes located in 1.4-Mb region was poorly understood. In the present study, we generated two mouse lines with different deletion sizes observed in 22q11.2DS; that is, *Del(1.4 Mb)/*+ and *Del(1.5 Mb)/*+ mice in inbred C57BL/6N background. We characterized ethological phenotypes of *Del(1.4 Mb)/*+ and *Del(1.5 Mb)/*+ mice and compared them with phenotypes of existing 22q11.2DS models including our previous *Del(3.0 Mb)/*+ mice.

Our open field test showed that *Del(1.4 Mb)/*+ mice exhibited lower locomotor activity in a novel environment. This hypoactive phenotype is consistent with that of *Del(3.0 Mb)/*+ mice [19]. On the other hand, the general locomotor activity of *Del(1.5 Mb)/*+ mice was normal. These results suggest that the deletion of 1.4-Mb region, but not the deletion of 1.5-Mb region, is responsible for the hypoactive phenotype. However, the *Lgdel/*+ and *Df(16)A*^+*/−*^ mice which are 22q11.2DS mouse models with the 1.5-Mb deletion displayed hyperactivity. This phenotypic discrepancy between our *Del(1.5 Mb)/*+ model and other 1.5-Mb deletion models may derive from different murine genetic backgrounds. The *Lgdel/*+ and *Df(16)A*^+*/−*^ mice are not congenic models [17, 33], while our *Del(1.5 Mb)/*+ model is a coisogenic strain generated by CRISPR/Cas9 system using C57BL/6N embryos. In line with this, the *Df(h22q11)/*+ model established in C57BL/6N congenic strain has displayed normal locomotion in the dark and bright conditioned open field task [44]. Regarding the anxiety-related behavior, *Crkl*^+*/−*^ mice spent less time in the margin area of the open field than *Crkl*^+*/*+^ mice for 30 min test period, suggesting that *Crkl* heterozygous mutation act as a protective factor for phobia-like behavior [43]. However, *Del(1.4 Mb)/*+ mice did not show the zone preference in open field arena for 10 min test period. This inconsistent results between *Del(1.4 Mb)/*+ and *Crkl*^+*/−*^ mice might be caused by different testing period in open field tests.

The impairment in basal sociability or facial recognition has been observed in 22q11.2DS patients [45–50]. In our experiments, sociability and social memory were apparently intact in *Del(1.4 Mb)/*+ mice, suggesting that social behavior is not affected by the deletion of genes within 1.4-Mb region. *Del(1.5 Mb)/*+ mice also showed apparently normal sociability and social memory. Interestingly, our previous study showed that the social recognition is impaired in *Del(3.0 Mb)/*+ mice [19]. Together, these results suggest that the impairment of social recognition requires multiple mutations both in 1.4-Mb and 1.5-Mb regions.

Our PPI analysis showed that the sensorimotor gating of *Del(1.4 Mb)/*+ mice were apparently intact. This result suggests that the 1.4-Mb deletion does not affect the sensorimotor gating. On the contrary, the reduced PPI was observed in *Del(1.5 Mb)/*+ mice, which is consistent with *Df1/*+, *Df(16)A*^+*/−*^, *Df(h22q11)/*+ and *Del(3.0 Mb)/*+ mice [17, 19, 30–32, 38, 39]. Therefore, genes located within the 1.5-Mb region is highly likely to be responsible for the defective sensorimotor gating observed in 22q11.2DS mouse models. In fact, *Dgcr8* located in 1.5-Mb region is a strong candidate gene for the impaired PPI. *Dgcr8*^+*/−*^ mice showed decreased PPI, which was rescued by administration of haloperidol [51]. However, in our study, haloperidol did not rescue the PPI impairment in *Del(3.0 Mb)/*+ mice. This inconsistent result could be attributable to different drug administration period. While our *Del(3.0 Mb)/*+ mice were treated with single-shot haloperidol 30 min before the test, *Dgcr8*^+*/−*^ mice have been continuously treated with haloperidol for 3–4 weeks [51], suggesting that continuous administration of haloperidol is required to rescue the PPI impairment instead of acute administration. On the other hand, the hearing loss arising from otitis media was observed in *Df1/*+ mice [52]. Therefore, the reduction of PPI might be caused by a hearing loss, and haloperidol administration was ineffective on PPI deficits in *Del(3.0 Mb)/*+ mice.

In our fear-conditioning test, contextual- and cued-dependent learning of *Del(1.4 Mb)/*+ mice was apparently normal, indicating that learning and memory are not affected by the 1.4-Mb deletion. On the other hand, *Del(1.5 Mb)/*+ mice, as expected, displayed the impairment in contextual- and cued-dependent learning, which is consistent with *Df1/*+ , *Df(16)A*^+*/−*^ and *Lgdel/*+ mice [17, 31–33, 40, 41]. These findings support the theory that fear memory impairment is caused by the deletion of 1.5-Mb region but not the deletion of 1.4-Mb region. Consistent with this, mice with the conditional deletion of *Dgcr8* within 1.5-Mb region in thalamic neurons have exhibited the associative fear memory deficits and the disrupted synaptic transmission at thalamic input to the lateral amygdala (LA) [41]. Hence, it is possible that the fear memory deficits of *Del(1.5 Mb)/*+ mice were caused by decreased *Dgcr8* expression.

EEG/EMG recording revealed that wakefulness and NREMS patterns were significantly disturbed in *Del(3.0 Mb)/*+ mice immediately after the start of dark period; wakefulness reduced and NREMS increased at ZT14 compared with WT littermates. *Del(3.0 Mb)/*+ mice managed to wake up at ZT12 just like WT littermates, indicating that their response to light stimulation during light-to-dark transition is intact, but *Del(3.0 Mb)/*+ mice are unable to maintain their wakefulness after that. On the other hand, this disturbance of hourly patterns during the first few hours immediately after the start of dark phase has not observed in behavioral activity-based circadian rhythm study of *Del(3.0 Mb)/*+ mice [19]. This discrepancy may arise from the differences of the recording environment and detectors between the EEG/EMG-based and behavioral activity-based experiments. Therefore, it is difficult to simply compare these results. In contrast to wakefulness and NREMS stages, there was no significant difference in the REMS pattern between *Del(3.0 Mb)/*+ and WT littermates. As for REMS regulation, *Goosecoid-like* (*Gscl*), a homeobox transcription factor, and *Dgcr14* are located within the 3.0-Mb region, and their absence is known to result in REMS disturbances [53]. *Gscl*^*−/−*^ mice concurrently lacked the expression of *Dgcr14* in the interpeduncular nucleus and showed abnormal REMS pattern [53]. Given that our *Del(3.0 Mb)/*+ mice have a heterozygous deletion of *Gscl* and *Dgcr14* gene with the other allele intact, it is reasonable to consider that these genes are haploinsufficient in the context of REMS regulation, thus REMS of *Del(3.0 Mb)/*+ mice stayed unaffected. On the other hand, our EEG spectral analysis showed reduction in theta power during REMS of *Del(3.0 Mb)/*+ mice, which is congruous with *Gscl*^*−/−*^ mice [53], suggesting that *Gscl* and *Dgcr14* are haploinsufficient in the context of theta power regulation during REMS.

In summary, we generated two novel mouse models mimicking 1.4-Mb or 1.5-Mb deletions seen in 22q11.2DS patients on pure C57BL/6 genetic background. These mice were comprehensively analyzed to determine the deletion regions responsible for psychiatric disorder-like phenotypes observed in *Del(3.0 Mb)/*+ mice. The hypoactive phenotype is caused by deletion of the genes in 1.4 Mb region, while genes within 1.5 Mb regions are responsible for impairment of sensorimotor gating and fear memory. The impairment of social recognition requires mutations both in 1.4 Mb and 1.5 Mb regions. We also found the disturbed wakefulness and NREMS cycles in *Del(3.0 Mb)/*+ mice. In conclusion, this study using our model mice shed light on the genetic contribution to the diverse symptoms of 22q11.2DS.

## Supplementary Information

**Additional file 1: Table S1.** sgRNA sequences for generating *Del(1.4 Mb)/*+ and *Del(1.5 Mb)/*+ mice

**Additional file 2: Table S2.** Single-stranded oligodeoxyribonucleotide (ssODN) sequences for generating *Del(1.4 Mb)/*+ and *Del(1.5 Mb)/*+ mice

**Additional file 3: Table S3.** Primer sequences for quantitative RT-PCR

**Additional file 4: Table S4.** The information of statistical methods and values

**Additional file 5: Table S5.** Efficiencies of generating *Del(1.4 Mb)/*+ and *Del(1.5 Mb)/*+ mice

**Additional file 6: Figure S1.** Effects of haloperidol administration on PPI deficits in *Del(3.0 Mb)/*+ mice.

## Data Availability

The datasets used and/or analyzed during the current study are available from the corresponding author on reasonable request.

## Abbreviations

22q11.2DS: 22Q11.2 deletion syndrome
ADHD: Attention deficit hyperactivity disorder
ANOVA: Analysis of variance
AP: Anteroposterior
bp: Base pair
Cas9: CRISPR-associated 9
cDNA: Complementary deoxyribonucleic acid
CGH: Comparative genomic hybridization
CNV: Copy number variation
CRISPR: Clustered regularly interspaced short palindromic repeats
dB: Decibel
DNA: Deoxyribonucleic acid
dT: Deoxythymidine
DV: Dorsoventral
EEG: Electroencephalogram
EMG: Electromyogram
IP: Interpeduncular nucleus
LCRs: Low copy repeats
ML: Mediolateral
mRNA: Messenger ribonucleic acid
mWM: Modified Whitten’s medium
NAHR: Non-allelic homologous recombination
NREMS: Non-rapid eye movement sleep
PAM: Protospacer adjacent motif
PCR: Polymerase chain reaction
PPI: Prepulse inhibition
REMS: Rapid eye movement sleep
RNA: Ribonucleic acid
RT: Reverse transcription
SEM: Standard error of mean
sgRNA: Single-guide ribonucleic acid
ssODN: Single-stranded oligodeoxyribonucleotide
TE: Tris-ethylenediaminetetraacetic acid buffer
WT: Wild-type
ZT: Zeitgeber time
