# Supplementary material for: Two novel mouse models mimicking minor deletions in 22q11.2 deletion syndrome revealed the contribution of each deleted region to psychiatric disorders
Source: Mol Brain. 2021 Apr 12;14:68. doi: 10.1186/s13041-021-00778-7 (PMC8042712; doi:10.1186/s13041-021-00778-7)
Supplement: Supplementary file 1 — Additional file 1: Table S1. sgRNA sequences for generating Del(1.4 Mb)/+ and Del(1.5 Mb)/+ mice [file 13041_2021_778_MOESM1_ESM.docx]

**Additional file 1**

**Additional Table S1.** sgRNA sequences for generating *Del(1.4Mb)/+* and *Del(1.5Mb)/+* mice.

| sgRNA | sequences (5'-3') |
| --- | --- |
| Pi4ka sgRNA_1 | CAGGACTGGGACTCGAGACGGGG |
| Pi4ka sgRNA_2 | ATGCGGCCCCACAGATCTGGAGG |
| Dgcr2 sgRNA_1 | CCCGAAGGGAGAACTCGTTGTGG |
| Dgcr2 sgRNA_2 | TCACCATTGTTTGGACCCAAAGG |
| Hira sgRNA_1 | GAGGAGGTCGCCTATTGTCCAGG |
| Hira sgRNA_2 | GGTAGAAGGAGTGGGCTAACAGG |

Underlined parts indicate PAM sequences.
