# Supplementary material for: Two novel mouse models mimicking minor deletions in 22q11.2 deletion syndrome revealed the contribution of each deleted region to psychiatric disorders
Source: Mol Brain. 2021 Apr 12;14:68. doi: 10.1186/s13041-021-00778-7 (PMC8042712; doi:10.1186/s13041-021-00778-7)
Supplement: Supplementary file 2 — Additional file 2: Table S2. Single-stranded oligodeoxyribonucleotide (ssODN) sequences for generating Del(1.4 Mb)/+ and Del(1.5 Mb)/+ mice [file 13041_2021_778_MOESM2_ESM.docx]

**Additional file 2**

**Additional Table S2.** Single-stranded oligodeoxyribonucleotide (ssODN) sequences for generating *Del(1.4Mb)/+* and *Del(1.5Mb)/+* mice.

| Deletion model | Bridging ssODN (5'-3') |  |
| --- | --- | --- |
| *Del(1.4Mb)/+* | GCCAGAGCCTGATGCTCCCTTGCAGGAGCACGGCCACCTCCATACTGATGCTGGCCTCCACAAAGGACTGTGCTTCCTTCTAGAGAAGGGGAGACACCTGGCTACCCTTTCAAGCTTCCA |  |
|  |  |  |
|  |  |  |
|  |  |  |
| *Del(1.5Mb)/+* | CCTTTTCCTGGTCCCAGGGGTTCAAGTTTGCAAAGCAAGTACTCTACTCCCGCACCACAATCCAGGGGCTGATGAATGAGTGAGGAACTATTCATGGTTCTCTAGGCCTCAAGTAGAAAG |  |
|  |  |  |
|  |  |  |
|  |  |  |
