# Supplementary material for: Two novel mouse models mimicking minor deletions in 22q11.2 deletion syndrome revealed the contribution of each deleted region to psychiatric disorders
Source: Mol Brain. 2021 Apr 12;14:68. doi: 10.1186/s13041-021-00778-7 (PMC8042712; doi:10.1186/s13041-021-00778-7)
Supplement: Supplementary file 3 — Additional file 3: Table S3. Primer sequences for quantitative RT-PCR [file 13041_2021_778_MOESM3_ESM.docx]

**Additional file 3**

**Additional Table S3.** Primer sequences for quantitative RT-PCR.

| Gene | Forward (5'-3') | Reverse (5'-3') |
| --- | --- | --- |
| *Hprt* | GGTTAAGCAGTACAGCCCCA | GTCTGGCCTGTATCCAACACT |
| *Actb* | CCTTCTTGGGTATGGAATCCTGT | TGGCATAGAGGTCTTTACGGATG |
| *Pi4ka* | GGCTGGGAACCAGACATCAA | CATCCATATAGGGGCGCACA |
| *Serpind1* | GCCATTGACCTGTTCAAGCA | GGGACGGTCGACAGTGAATC |
| *Snap29* | AACCTAGATGAGCTGTCCGTG | TGGTTGTCAGTCGGTCAAGG |
| *Crkl* | ACGTGCTAGATAACCGGCTG | TTTCAGCTGAGGCTGGGATG |
| *Aifm3* | CTTGCCTGGAGGAACAATCG | CATGGCAGTCCACAGATAGGG |
| *Lztr1* | GGCTTACTGCAAGCAAAACCT | GCAGCTTAGAGACCTTGGTGA |
| *Thap7* | CTGGTGGGAATCAGTGGGTA | TGTTCGACGCAACTTGGAGA |
| *Lrrc74b* | TGACCTGGCAGGAGAGATACT | CAGACCTCGCAGGTGATTCC |
| *P2rx6* | GAAGTTCGCGCTCATCCCTA | GGGGCTCTTGCCTCTTCATA |
| *Slc7a4* | CTGGTCGGACTTGTCGTGT | CTCCCGGACTGGTGATTGAC |
| *Smpd4* | CCGGAGCTACGAAATCACCA | TCTGGCCTGCAAATCTACGG |
| *Ccdc74a* | GAGATCGAGCACCTGAAGCG | GAGAGTTGGCGGACATCGTG |
| *Med15* | CACCTATCGTGTCGCCAGTG | ACCTTGCAGCACATTGGGTA |
| *Klhl22* | CCCCACTCAAGAAGGAGGTAT | TATGCCACGTGTTGCTTCCT |
| *Scarf2* | GAAGGCGCCTCAACGTTTTT | ATGGGCCACTACGACTTTGG |
| *Car15* | CATGCGCAGGTAGTCCAGTT | GGGGCGGAAATTACTCGTGA |
| *Dgcr2* | TTCCATTTCCACGACCCTCC | CTCAAAGGCATCGTCATCTGC |
| *Tssk1* | CGGTCTGAGTCAAAACCCCA | GGCTGCAAGAGGCTCACTAA |
| *Dgcr14* | ACTTGGCCAGTCTTACTCCCA | GAGGTGGGAAGATCGTGCTG |
| *Slc25a1* | TGCAGCCAGTGTCTTTGGAA | GTAGAATGCCTTTGGCCCCT |
| *Dgcr6* | CGAACACCGAGTGCTCAGAC | ATCCGATGTTCCATGGCCT |
| *Prodh* | TTTATGCCCAAGGCGGGATT | TTGGGGTACAGGAAGTCCCA |
| *Rtn4r* | GAGCTTCCAGTCATGCCGAA | GGTCCACGACATGAAGCTGT |
| *Zdhhc8* | AGAAACCTCTGGACCTGGGA | TGATAGGGCACTCTCAGCAC |
| *Ranbp1* | CTTCCTAAATGCTGAGAATGCAC | CCTCCCTCACTGAAAGGGC |
| *Trmt2a* | TAAGGTGATTCTGGCCATCCG | CCTGCAGAGGTCCACAAAGTT |
| *Dgcr8* | GCGCGGGTGGTGTAAGAATAA | TGCTGCTCTCACGACCATAC |
| *Arvcf* | ACGAAGTCACGCTTCCAGTC | TTGACTTCTCCCCATCAAGGC |
| *Comt* | ATTGTGGCTACTCAGCCGTG | CCCGATGAGGATGGAAACTTTG |
| *Txnrd2* | GGATCAAGTGTGGGGCTTCA | GCAACCAGTCACAGTAGGCT |

*(Table continued on next page)*

*(Continued)*

| *Gnb1l*  *Gp1bb*  *Sept5* | TCCCTGCAGGTGAAGAAGACT  AGTGATGGAACAGCCCAGTC  AACATGCTCATCCGCACTCA | AACACACGGATGCGATGGTC  TTTGGCAAAGTCGGGTGGTA  CTGGGTGAGTTTGCTGGTCA |
| --- | --- | --- |
| *Cldn5* | GTTAAGGCACGGGTAGCACT | TACTTCTGTGACACCGGCAC |
| *Cdc45* | GAGGGCACTCCAGATGTCAC | GCATCGTCGATTCTTTGTCGAG |
| *Ufd1l* | TTGAAGAGGATGAAGCTGGAGG | CAGTAACTGTAAGCCAGGTGC |
| *Mrpl40* | GCAGCAAAGGATCGCTTGAA | CTCCTGAGGTCGCTGTCTTG |
| *Hira* | CCTCCAACTCTGGAAGGCAAG | AGGAGGAGCCAGTGACGATA |
