# Supplementary material for: Two novel mouse models mimicking minor deletions in 22q11.2 deletion syndrome revealed the contribution of each deleted region to psychiatric disorders
Source: Mol Brain. 2021 Apr 12;14:68. doi: 10.1186/s13041-021-00778-7 (PMC8042712; doi:10.1186/s13041-021-00778-7)
Supplement: Supplementary file 4 — Additional file 4: Table S4. The information of statistical methods and values [file 13041_2021_778_MOESM4_ESM.docx]

**Additional file 4**

**Additional Table S4.** The information of statistical methods and values.

| Figure 3a | Two-tailed Welch's *t*-test. *t*_21.67_ = 3.719. ***p* = 0.0012.  WT: n = 12, *Del(1.4Mb)/+*: n = 12. |
| --- | --- |
| Figure 3b | Two-tailed Welch's *t*-test. *t*_21.79_ = 0.3554. *p* = 0.7257.  WT: n = 12, *Del(1.4Mb)/+*: n = 12. |
| Figure 3c | Two-tailed Welch's *t*-test. *t*_20.82_ = 2.033. *p* = 0.0550.  WT: n = 11, *Del(1.5Mb)/+*: n = 12. |
| Figure 3d | Two-tailed Welch's *t*-test. *t*_17.28_ = 1.478. *p* = 0.1575.  WT: n = 11, *Del(1.5Mb)/+*: n = 12. |
| Figure 4a | 1–4 trial: Two-way repeated measures ANOVA. Genotype: *F*_1,22_ = 0.2458, *p* = 0.6249. Trial: *F*_3,66_ = 28.9, *p* < 0.0001. Interaction: *F*_3,66_ = 1.001, *p* = 0.3980. 5 trial: Two-tailed Welch's t-test. *t*_16.09_ = 1.092. *p* = 0.291.  WT: n = 12, *Del(1.4Mb)/+*: n = 12. |
| Figure 4b | 1–4 trial: Two-way repeated measures ANOVA. Genotype: *F*_1,21_ = 0.4986, *p* = 0.4879. Trial: *F*_3,63_ = 11.1, *p* < 0.0001. Interaction: *F*_3,63_ = 0.8155, *p* = 0.4901. 5 trial: Two-tailed Welch's *t*-test. *t*_18.2_ = 0.1601. *p* = 0.8746.  WT: n = 11, *Del(1.5Mb)/+*: n = 12. |
| Figure 5a | Two-way repeated measures ANOVA. Genotype: *F*_1,22_ = 0.9059, *p* = 0.3516. Prepulse intensity: *F*_3,66_ = 33.71, *p* < 0.0001. Interaction: *F*_3,66_ = 0.1952, *p* = 0.8993. WT: n = 12, *Del(1.4Mb)/+*: n = 12. |
| Figure 5b | Two-tailed Welch's *t*-test. *t*_20.43_ = 0.006398. *p* = 0.995.  WT: n = 12, *Del(1.4Mb)/+*: n = 12. |
| Figure 5c | Two-way repeated measures ANOVA. Genotype: *F*_1,21_ = 5.159, **p* = 0.0338. Prepulse intensity: *F*_3,63_ = 47.78, *p* < 0.0001. Interaction: *F*_3,63_ = 0.2333, *p* = 0.8728. WT: n = 11, *Del(1.5Mb)/+*: n = 12. |

*(Table continued on next page)*

*(Continued)*

| Figure 5d | Two-tailed Welch's *t*-test. *t*_17.56_ = 1.13. *p* = 0.2738.  WT: n = 11, *Del(1.5Mb)/+*: n = 12. |
| --- | --- |
| Figure 6a | Two-tailed Welch's *t*-test. Context: *t*_21.1_ = 0.7505. *p* = 0.4612. Pre tone: *t*_21.86_ = 1.169. *p* = 0.2551. Tone: *t*_20.03_ = 0.5174. *p* = 0.6106. WT: n = 12, *Del(1.4Mb)/+*: n = 12. |
| Figure 6b | Two-tailed Welch's *t*-test. Context: *t*_44.8_ = 3.875. ****p* = 0.0003. Pre tone: *t*_44.16_ = 1.352. *p* = 0.1832. Tone: *t*_44.71_ = 4.997. ****p* < 0.0001. WT: n = 11, *Del(1.5Mb)/+*: n = 12. |
| Figure 7a | Two-way repeated measures ANOVA. Genotype: *F*_1,23_ = 6.672, **p =* 0.0166. Zeitgeber time: *F*_23,529_ = 56.15, *p* < 0.0001. Interaction: *F*_23,529_ = 1.784, *p* = 0.0143. Bonferroni's multiple comparisons test.  ZT14: ***p* = 0.0015. WT: n = 9, *Del(3.0Mb)/+*: n = 16. |
| Figure 7b | Two-way repeated measures ANOVA. Genotype: *F*_1,23_ = 8.498, ***p =* 0.0078. Zeitgeber time: *F*_23,529_ = 49.36, *p* < 0.0001. Interaction: *F*_23,529_ = 1.586, *p* = 0.0416. Bonferroni's multiple comparisons test.  ZT14: ****p* = 0.0008. WT: n = 9, *Del(3.0Mb)/+*: n = 16. |
| Figure 7c | Two-way repeated measures ANOVA. Genotype: *F*_1,23_ = 2.294, *p =* 0.1435. Zeitgeber time: *F*_23,529_ = 48.81, *p* < 0.0001. Interaction: *F*_23,529_ = 2.393, *p* = 0.0003. WT: n = 9, *Del(3.0Mb)/+*: n = 16. |

*(Table continued on next page)*

*(Continued)*

| Figure 7d | Welch's *t*-test. Wake 24h: *t*_8.712_ = 1.996. *p* = 0.0781. NREM 24h: *t*_8.996_ = 2.29. **p* = 0.0478. REM 24h: *t*_15.19_ = 1.466. *p* = 0.1630. Wake_L: *t*_10.43_ = 1.944. *p* = 0.0793. NREM_L: *t*_12.2_ = 2.633. **p* = 0.0216. REM_L: *t*_15.61_ = 1.935. *p* = 0.0713. Wake_D: *t*_8.806_ = 1.847. *p* = 0.0986. NREM_D: *t*_8.963_ = 1.978. *p* = 0.0794. REM_D: *t*_15.18_ = 0.2168. *p* = 0.8313. WT: n = 9, *Del(3.0Mb)/+*: n = 16. |
| --- | --- |
| Figure 7e | Welch's *t*-test. Wake 24h: *t*_9.369_ = 1.374. *p* = 0.2014. NREM 24h: *t*_22.71_ = 1.139. *p* = 0.2667. REM 24h: *t*_14.06_ = 1.793. *p* = 0.0945. Wake_L: *t*_11.61_ = 1.436. *p* = 0.1774. NREM_L: *t*_22.88_ = 0.6158. *p* = 0.5441. REM_L: *t*_16.95_ = 1.443. *p* = 0.1673. Wake_D: *t*_8.486_ = 1.485. *p* = 0.1736. NREM_D: *t*_21.45_ = 2.043. *p* = 0.0535. REM_D: *t*_12.18_ = 2.175. *p* = 0.0500. WT: n = 9, *Del(3.0Mb)/+*: n = 16. |
| Figure 7f | Welch's *t*-test. 24 h: *t*_22.69_ = 0.3545. *p* = 0.7263. Light: *t*_22.35_ = 0.4509. *p* = 0.6564. Dark: *t*_21.74_ = 0.06487. *p* = 0.9489. WT: n = 9, *Del(3.0Mb)/+*: n = 16. |
| Figure 7g | Welch's *t*-test. 24 h: *t*_14.5_ = 1.709. *p* = 0.4896. Light: *t*_18.83_ = 0.03212. *p* = 0.9747. Dark: *t*_12.39_ = 0.2673. *p* = 0.7936. WT: n = 9, *Del(3.0Mb)/+*: n = 16. |

*(Table continued on next page)*

*(Continued)*

| Figure 7h | Welch's *t*-test. Wakefulness → NREMS: *t*_15.36_ = 0.7609. *p* = 0.4583. Wakefulness → REMS: *t*_8_ = 1.414. *p* = 0.1950. NREMS → Wakefulness: *t*_16.29_ = 0.4326. *p* = 0.6709. NREMS → REMS: *t*_17.86_ = 0.6444. *p* = 0.5275. REMS → Wakefulness: *t*_22.92_ = 0.9152. *p* = 0.3696. REMS → NREMS: *t*_14.75_ = 0.05409. *p* = 0.9576. WT: n = 9, *Del(3.0Mb)/+*: n = 16. |
| --- | --- |
| Figure 7i | Two-way repeated measures ANOVA. Genotype: *F*_1,23_ = 0.005049, *p =* 0.9440. Frequency: *F*_29,667_ = 643.5, *p* < 0.0001. Interaction: *F*_29,667_ = 0.9918, *p* = 0.4795. WT: n = 9, *Del(3.0Mb)/+*: n = 16. |
| Figure 7j | Two-way repeated measures ANOVA. Genotype: *F*_1,23_ = 0.04029, *p =* 0.8427. Frequency: *F*_29,667_ = 874.3, *p* < 0.0001. Interaction: *F*_29,667_ = 1.521, *p* = 0.0404. WT: n = 9, *Del(3.0Mb)/+*: n = 16. |
| Figure 7k | Two-way repeated measures ANOVA. Genotype: *F*_1,23_ = 4.909, **p =* 0.0369. Frequency: *F*_29,667_ = 480.6, *p* < 0.0001. Interaction: *F*_29,667_ = 2.99, *p <* 0.0001. Bonferroni's multiple comparisons test. 7 Hz: ***p* = 0.0055; 8 Hz: ****p <* 0.0001; 9 Hz: ****p* = 0.0004. WT: n = 9, *Del(3.0Mb)/+*: n = 16. |

*(Table continued on next page)*

*(Continued)*

| Additional Figure S1a | Two-way repeated measures ANOVA. Genotype: *F_3,92_* = 12.67, ****p* < 0.0001. Prepulse intensity: *F_3,276_* = 202.8, *p* < 0.0001. Interaction: *F_9,276_* = 1.069, *p* = 0.3860. Bonferroni's multiple comparisons test. [69 dB] WT SAL vs. WT HAL: ***p* = 0.0096. *Del(3.0Mb)/+* SAL vs. WT HAL: ****p* = 0.001. WT HAL vs. *Del(3.0Mb)/+* HAL: ***p* = 0.0036. [73 dB] WT SAL vs. WT HAL: ***p* = 0.0164. *Del(3.0Mb)/+* SAL vs. WT HAL: ****p* < 0.0001. WT HAL vs. *Del(3.0Mb)/+* HAL: ****p* = 0.0006. [77 dB] WT SAL vs. WT HAL: **p* = 0.0254. *Del(3.0Mb)/+* SAL vs. WT HAL: ****p* < 0.0001. WT HAL vs. *Del(3.0Mb)/+* HAL: ***p* = 0.0022. [81 dB] WT SAL vs. *Del(3.0Mb)/+* SAL: ***p* = 0.008. *Del(3.0Mb)/+* SAL vs. WT HAL: ****p* < 0.0001. WT HAL vs. *Del(3.0Mb)/+* HAL: ****p* = 0.0002. WT SAL: n = 24, *Del(3.0Mb)/+* SAL: n = 24, WT HAL: n = 25, *Del(3.0Mb)/+* HAL: n = 23. |
| --- | --- |
| Additional Figure S1b | Kruskal-Wallis test. ****p* = 0.0007. Dunn's multiple comparisons post hoc test. WT SAL vs. *Del(3.0Mb)/+* SAL: *p* = 0.0610. WT SAL vs. WT HAL: *p* > 0.9999. WT SAL vs. *Del(3.0Mb)/+* HAL: *p* = 0.9835. *Del(3.0Mb)/+* SAL vs. WT HAL: ****p* = 0.0007. *Del(3.0Mb)/+* SAL vs. *Del(3.0Mb)/+* HAL: *p* > 0.9999. WT HAL vs. *Del(3.0Mb)/+* HAL: **p* = 0.0470. WT SAL: n = 24, *Del(3.0Mb)/+* SAL: n = 24, WT HAL: n = 25, *Del(3.0Mb)/+* HAL: n = 23. |
