# Supplementary material for: Two novel mouse models mimicking minor deletions in 22q11.2 deletion syndrome revealed the contribution of each deleted region to psychiatric disorders
Source: Mol Brain. 2021 Apr 12;14:68. doi: 10.1186/s13041-021-00778-7 (PMC8042712; doi:10.1186/s13041-021-00778-7)
Supplement: Supplementary file 5 — Additional file 5: Table S5. Efficiencies of generating Del(1.4 Mb)/+ and Del(1.5 Mb)/+ mice [file 13041_2021_778_MOESM5_ESM.docx]

**Additional file 5**

**Additional Table S5.** Efficiencies of generating *Del(1.4Mb)/+* and *Del(1.5Mb)/+* mice.

| Deletion models | Embryos injected^a^ | Transferred^b^  (%: b/a) | Pups born^c^  (%: c/b) | Weaning^d^  (%: d/c) | Desired mutant^e^ (%: e/d) |
| --- | --- | --- | --- | --- | --- |
| *Del(1.4Mb)/+* | 838 | 432 (51.6%) | 23 (5.3%) | 14 (60.9%) | 6 (42.9%) |
| *Del(1.5Mb)/+* | 641 | 452 (70.5%) | 13 (2.9%) | 10 (76.9%) | 2 (20.0%) |

Pups include mice died before weaning.
