# Supplementary material for: Two novel mouse models mimicking minor deletions in 22q11.2 deletion syndrome revealed the contribution of each deleted region to psychiatric disorders
Source: Mol Brain. 2021 Apr 12;14:68. doi: 10.1186/s13041-021-00778-7 (PMC8042712; doi:10.1186/s13041-021-00778-7)
Supplement: Supplementary file 6 — Additional file 6: Figure S1. Effects of haloperidol administration on PPI deficits in Del(3.0 Mb)/+ mice. [file 13041_2021_778_MOESM6_ESM.docx]

**Additional file 6**


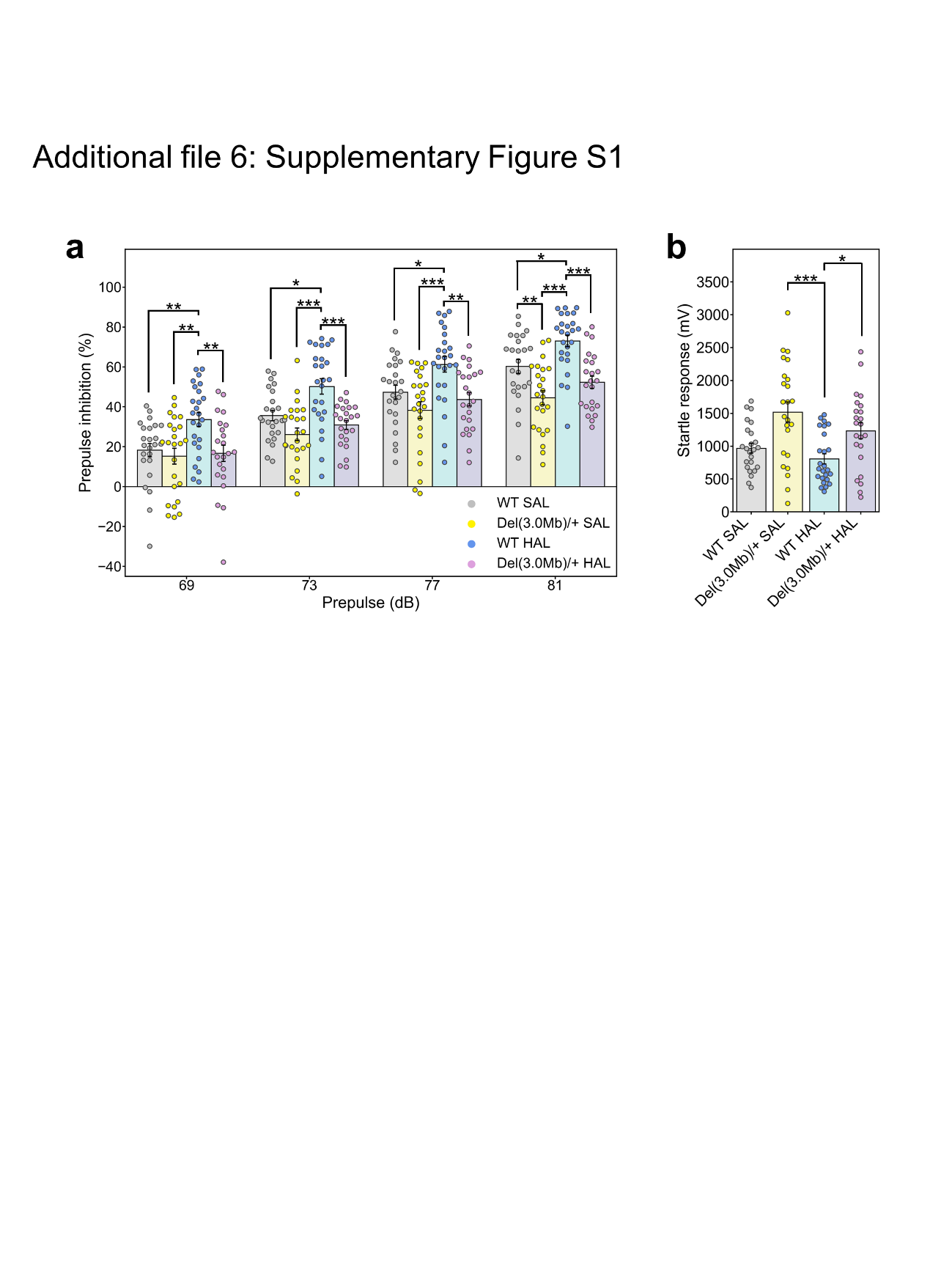


**Additional Figure S1. Effects of haloperidol administration on PPI deficits in *Del(3.0Mb)/+* mice**

(a) Percentage of PPI in *Del(3.0Mb)/+* mice which were administrated saline or haloperidol (0.3 mg/kg, i.p.) 30 min before the test. SAL, saline administrated group; HAL, haloperidol administrated group. (b) Measurement of acoustic startle response to the 120-dB startle stimulus. Data are expressed as mean ± SEM (n = 24 for WT SAL; n = 24 for *Del(3.0Mb)/+* SAL; n = 25 for WT HAL; n = 23 for *Del(3.0Mb)/+* HAL). **p* < 0.05, ** *p* < 0.01, *** *p* < 0.001.
